# Effect of variety and processing method on functional properties of traditional sweet potato flour (“elubo”) and sensory acceptability of cooked paste (“amala”)

**DOI:** 10.1002/fsn3.161

**Published:** 2014-08-07

**Authors:** Ganiyat Fetuga, Keith Tomlins, Folake Henshaw, Michael Idowu

**Affiliations:** 1 Department of Food Science and Technology, Federal University of AgricultureAbeokuta, Nigeria; 2 Food and Markets Department, Natural Resources Institute, University of GreenwichMedway, Kent, United Kingdom

**Keywords:** “Amala”, “elubo”, processing, quality, sweet potato flour, variety

## Abstract

“Amala” is a generic term in Nigeria, used to describe a thick paste prepared by stirring flour (“elubo”) from yam, cassava or unripe plantain, in hot water, to form a smooth consistency. In order to overcome its high perishability and increase the utilization of sweet potato roots, three varieties of sweet potato roots were processed into flour using two methods. The interactive effect of variety and the processing method had a significant effect (*P* < 0.05) on all the functional properties of the flour except yellowness, setback viscosity, and peak time. Acceptable sweet potato “amala” with average sensory acceptability score of 7.5 were obtained from yellow-fleshed varieties irrespective of the processing method. Flour that produced acceptable “amala” were characterized by lower values of protein (2.20–3.94%), fiber (1.30–1.65%), total sugar (12.41–38.83 *μ*g/mg), water absorption capacity (168–215 g/100 g), water solubility (8.29–14.65%), swelling power (0.52–0.82 g/g), and higher peak time (6.9–8.7 min).

## Introduction

Sweet potato [Ipomoea batatas L. (Lam.)] is an important staple food in many of the developing countries of the tropics and sub-tropics. Several varieties exist with a wide range of skin and flesh color, from white to yellow-orange and deep purple (Woolfe 1992; CIP 2013). Nigeria is currently the third largest producer of sweet potato in the world with a production of 2.12 million metric tonnes (FAOSTAT 2011). Sweet potato has been recognized as having an important role to play in improving household and national food security, health, and livelihoods of poor families in sub-Saharan Africa (Low et al. 2009). Sweet potato produces high yield even under marginal conditions (Horton et al. 1989). Despite its high carbohydrate content, sweet potato has a low glycemic index, indicating low digestibility of the starch (ILSI 2008). It is the only starchy staple with appreciable amounts of*β*-carotene (especially the orange-fleshed variety), ascorbic acid and amino acid lysine that is deficient in cereal-based diet like rice (Bradbury et al. 1985; Bradbury and Singh 1986a,b; Bradbury and Holloway 1988). In spite of having many desirable traits, sweet potato is still underutilized in Nigeria. The roots are difficult to handle and store due to its bulkiness and high perishability. In Nigeria, particularly the south western region, root and tuber crops such as yam and cassava are usually processed into flour known as “elubo” using traditional methods of parboiling in water or soaking followed by drying. This is to overcome the high perishability of fresh forms and the seasonal nature of their production. The traditional flour, “elubo”, is used to make a cooked paste meal known as “amala”. Extensive research on yam and cassava “elubo” is available in literature (Ige and Akintunde 1981; Oyewole and Odunfa 1988; Akissoe et al. 2001, 2003; Mestres et al. 2004; Babajide et al. 2006; Nwabueze and Odunsi 2007). Expanding utilization and market opportunities for sweet potato include producing products adapted to consumer preferences. Information on sweet potato “elubo” is limited. Tewe et al. (2003) had earlier reported that sweet potato is processed into “elubo”. A recent study also confirmed that sweet potato is processed into “elubo” but the production is still at the household level compared to the commercial scale of yam and cassava “elubo” (Fetuga et al. 2013). The study also reported that sweet potato “elubo” is produced from either of the two methods; parboiling or soaking in cold water (27–28°C), as applied to yam and cassava, respectively. The different methods were reported to result in varying sensory properties of “amala”, with consumers differing in their preferences (Fetuga et al. 2013). In view of the need for commercial production of sweet potato “elubo”, an understanding of the effect of processing variables such as variety and the processing method on properties of sweet potato “elubo” and sensory acceptability of its “amala” is required for quality control purposes. Therefore, this study was conducted to determine the effect of variety and the processing method on the chemical, functional, and pasting properties of sweet potato “elubo” and acceptability of its’ “amala”.

## Materials and Methods

### Sweet potato roots

Three varieties of sweet potato roots were used; a yellow-fleshed variety (“anoma pupa”) from Nigeria was obtained from a local market in Offa, Kwara State. The other two varieties were yellow-fleshed (variety not indicated), product of Uganda, and orange-fleshed (Dri-Pak brand), from America, both of which were obtained from Spitafields market in London, United Kingdom.

### Production of sweet potato flour (“elubo”)

The Nigerian variety was processed into “elubo” using each of the two traditional methods; parboiling and soaking in water, followed by sun drying and milling. The Ugandan and American varieties were processed into “elubo” with slight modifications to the traditional process, which involved using slices of 10 mm thickness instead of chunks of between 50–60 mm, and drying in a dehydrator at 52°C for 48 h instead of sun drying. The modifications were done for quality control purposes by using uniform thickness and controlled drying conditions expected for commercial production. Sweet potato “elubo” from the Nigerian variety served as reference.

### Preparation of sweet potato “amala”

Sweet potato “amala” was prepared by adding 200 g of “elubo” to 200 mL of boiling water. The “amala” was stirred manually with a wooden spoon over a low flame until a smooth consistency was attained. Portions of the “amala” were scooped and wrapped in polyethylene films and kept in a food flask until ready to serve.

### Chemical properties

The moisture, protein, crude fat, crude fiber, and ash contents of the “elubo” were determined using the methods of AOAC (2000). Carbohydrate was determined by difference according to James (1995). Starch was determined using the Phenol-sulfuric acid method of Dubois et al. (1956). Amylose was determined following the rapid colorimetric method of Williams et al. (1970). Fructose, glucose, sucrose, and total sugar were determined by reverse-phase high performance liquid chromatography using Agilent Zorbax NH2 guard colum at a flow rate of 2 mL/min, a column temperature of 30°C and an injection volume of 5 *μ*L. The solvent used was a mixture of 75% acetonitrile and 25% water. The detector was a refractive index type while the data system was Agilent EZChrom Elite version. The pH and total titratable acidity (TTA) were determined according to Pearson (1976).

### Hunter lab colour coordinates

The color of the “elubo” was measured using a Minolta CR-210 chromometer and the values expressed asL* (lightness),a* (redness),b* (yellowness). Brown index was calculated as (100 − L*) as described by Akissoe et al. (2004).

### Functional properties

Water absorption capacity (WAC) was determined by the method of Soluski et al. (1976) as described by Akubor (1997). Swelling power (SWP) and water solubility (WS) of the flour was determined as described by Aina et al. (2009).

### Pasting properties

The pasting properties were determined using a Rapid Visco Analyzer (RVA) (Newport Scientific, Australia. Three grams of the sample (14% moisture basis) and 25 mL of distilled water were used. A programmed heating and cooling cycle was used at constant shear rate, where the slurry was held at 50°C for 1 min, heated to 95°C within 7.5 min, and then held at 95°C for 5 min. It was subsequently cooled to 50°C within 8.5 min and held at 50°C for 2 min, while maintaining a rotation speed of 160 rpm. Total cycle time was 23 min.

### Consumer acceptability tests

One hundred consumers (Lyon et al. 1992) comprising undergraduate students of Federal University of Agriculture, Abeokuta, Ogun State, Nigeria, were asked to score the overall acceptability of the samples of sweet potato “amala” using a 9-point hedonic scale ranging from 1 “Dislike extremely” to 9 “Like extremely”.

### Statistical analysis

A multivariate General Linear Model (GLM) analysis was performed to determine the individual and interactive effects of the treatments (variety, pretreatment, and drying methods) on the attributes measured. Significant effects were established at*P* < 0.05, 0.01 and 0.001 levels. Pearson's correlation coefficient among the quality attributes was calculated. Statistical package used was SPSS Version 17.0 (SPSS Inc., Chicago, IL).

## Results

### Chemical properties

The moisture content of “elubo” ranged from 4.83% to 10.41% (Table1). “Elubo” from the Nigerian variety had high moisture content of 9.42% and 10.41%, for soaking and parboiling methods, respectively. The parboiling method gave a significantly higher (*P* < 0.05) moisture content compared to the soaking method, for the American orange-fleshed and Nigerian yellow-fleshed variety. “Elubo” from the orange-fleshed variety had significantly higher (*P* < 0.05) protein, fiber, and fat content (Table1) relative to other varieties but the lowest carbohydrate. The soaking method gave significantly higher (*P* < 0.05) carbohydrate values relative to parboiling for orange-fleshed and Nigerian yellow-fleshed varieties. Significantly lower (*P* < 0.05) fiber values were obtained for “elubo” from the soaking method relative to parboiling for all the varieties. Both variety and processing method had a significant effect (*P* < 0.001) on the moisture, fiber, fat, and carbohydrate content of sweet potato “elubo”.

**Table 1:** Proximate composition of traditional sweet potato flour (“elubo”) as affected by variety and the processing method.

Variety	Processing method	Moisture (%)	Protein (%)	Ash (%)	Fiber (%)	Fat (%)	Carbohydrate (%)
UYF	Soak in water	5.16ab	3.94b	1.82a	1.36b	0.07a	87.68d
	Parboil	4.83a	3.79a	1.94b	1.65c	0.25b	87.55d
AOF	Soak in water	5.43b	4.52d	2.07c	4.39d	1.07d	82.51b
	Parboil	9.65c	4.60de	1.87ab	5.37e	1.61e	76.90a
NYF	Soak in water	9.42c	2.33e	1.95b	0.73a	0.58c	84.98c
	Parboil	10.41d	2.20c	2.07c	1.30b	0.61c	83.42b
Effects							
Variety (V)		***	***	*	***	***	***
Processing method (P)		***	**	NS	***	***	***
V × P		***	**	**	***	***	***

Values followed by the same alphabet are not significantly different (*P* > 0.05). UYF, Ugandan Yellow-fleshed; AOF, American Orange-fleshed; NYF, Nigerian Yellow-fleshed; NS, not significant.

^*^, ^*^^*^, ^*^^*^^*^Significant effects at*P* < 0.05, 0.01, 0.001 respectively.

Variety and the processing method had a significant effect on starch, amylose, and sugars in sweet potato “elubo” (Table2). For all the varieties, fructose, sucrose, total sugar, and TTA of flour from the parboiling method were generally lower. Sucrose was the main sugar contributing to the total sugar in sweet potato “elubo”. The total sugar in “elubo” from orange-fleshed was between 6 and 28 times higher than in yellow-fleshed varieties.

**Table 2:** Chemical properties of traditional sweet potato flour (“elubo”) as affected by variety and the processing method.

Variety	Processing method	Starch (%)	Amylose (%)	Fructose (*μ*g /mg)	Glucose (*μ*g/mg)	Sucrose (*μ*g/mg)	Total sugar (*μ*g/mg)	pH	TTA (%)
UYF	Soak in water	70.30f	14.88c	3.49b	2.35a	33.00b	38.83b	5.49b	0.09b
	Parboil	59.86c	16.79d	0.28a	8.47ab	27.92b	36.67b	5.17a	0.07a
AOF	Soakin water	60.93d	18.92f	16.24e	16.10bc	201.24e	233.58e	5.33ab	0.26e
	Parboil	41.25a	12.23a	9.11d	21.69c	142.05d	172.85d	5.47ab	0.12d
NYF	Soak in water	59.74b	17.63e	5.71c	13.86bc	19.31ab	38.87b	5.21ab	0.11bc
	Parboil	60.27d	13.78b	4.77c	1.94a	5.70a	12.41a	5.40ab	0.07a
Effects									
Variety (V)		***	**	***	**	***	***	NS	***
Processing method (P)		***	***	***	NS	**	**	NS	***
V × P		***	***	***	*	**	**	*	***

Values followed by the same alphabet are not significantly different (*P* > 0.05). UYF, Ugandan Yellow-fleshed; AOF, American Orange-fleshed; NYF, Nigerian Yellow-fleshed; NS, not significant.

^*^, ^*^^*^, ^*^^*^^*^Significant effects at*P* < 0.05, 0.01, 0.001 respectively.

### Functional properties

“Elubo” from the Ugandan variety had the lowest brown index (100 − L*) values (1.73–1.95); although they were prepared from a yellow-fleshed variety similar to the Nigerian variety, the brown index were significantly different (Table3). Significantly higher (*P* < 0.05) redness (a*) and yellowness (b*) values were obtained for “elubo” from the orange-fleshed variety. Parboiling gave a significantly lower (*P* < 0.05) brown index for the Nigerian yellow-fleshed variety, while it was significantly higher (*P* < 0.05) for the orange-fleshed. The processing method did not have a significant effect (*P* > 0.05) on the (100 − L*),a*,b* values for the Ugandan variety. Variety was more significant on color parameters than the processing method.

**Table 3:** Hunter color parameters of traditional sweet potato flour (“elubo”) as affected by variety and the processing method.

Variety	Processing method	100 − L^*^	a^*^	b^*^
UYF	Soak in water	1.95d	0.07a	13.33a
	Parboil	1.73d	−0.01a	12.47a
AOF	Soak in water	16.82b	15.28c	33.28c
	Parboil	25.63a	20.04d	34.09c
NYF	Soak in water	16.35b	2.35b	15.36b
	Parboil	12.08c	2.35b	13.30a
Effects				
Variety (V)		***	***	***
Processing method (P)		NS	**	NS
V × P		**	**	NS

Values followed by the same alphabet are not significantly different (*P* > 0.05). UYF, Ugandan Yellow-fleshed; AOF, American Orange-fleshed; NYF, Nigerian Yellow-fleshed;L^*^, lightness;a^*^, redness;b^*^, yellowness; (100 − L^*^), brown index; NS, not significant.

^*^, ^*^^*^, ^*^^*^^*^Significant effects at*P* < 0.05, 0.01, 0.001 respectively.

“Elubo” from the American orange-fleshed variety had the highest WAC, WS, and SWP (Table4), which were significant (*P* < 0.05). Although parboiling resulted in a significant increase in WAC of between 22% (Ugandan) and 61% (orange-fleshed), there was no difference for the Nigerian variety. The processing method also did not show a significant difference in WS for the Ugandan variety. Generally, for all the three varieties, “elubo” from the parboiling method had significantly higher (*P* < 0.05) SWP than those from the soaking method. The WAC, WS, and SWP of sweet potato flour were all significantly affected (*P* < 0.01) by variety and the processing method.

**Table 4:** Functional properties of traditional sweet potato flour (“elubo”) as affected by variety and the processing method.

Variety	Processing method	WAC	WS	SWP
UYF	Soak in water	167.50a	13.94b	0.52a
	Parboil	215.00b	14.65b	0.82bc
AOF	Soak in water	272.50c	52.40d	0.89c
	Parboil	702.50d	44.62c	3.71d
NYF	Soak in water	196.50b	14.56b	0.66ab
	Parboil	196.50b	8.29a	0.79bc
Effects				
Variety (V)		***	***	***
Processing method (P)		***	**	***
V × P		***	*	***

Values followed by the same alphabet are not significantly different (*P* > 0.05). UYF, Ugandan Yellow-fleshed; AOF, American Orange-fleshed; NYF, Nigerian Yellow-fleshed; WAC, water absorption capacity; WS, water solubility; SWP, swelling power; NS, not significant.

^*^, ^*^^*^, ^*^^*^^*^Significant effects at*P* < 0.05, 0.01, 0.001 respectively.

### Pasting properties

For all the varieties, parboiled samples had higher paste viscosities than soaking in water (Table5). Although the range of peak temperature (PTp) was (50.69–78.58°C), “elubo” produced from the orange-fleshed variety by soaking in water, did not form a paste, and was also associated with very low viscosity values. Variety and the processing method had a significant effect (*P* < 0.001) on peak viscosity (PV), trough (T) and the final viscosity (FV), however, the interaction between the two factors did not show a significant effect (*P* > 0.05) on setback viscosity (SBV) and peak time (PT). PT was also not significantly affected (*P* > 0.01) by the variety and the processing method or the interaction between both treatments.

**Table 5:** Pasting properties of traditional sweet potato flour (“elubo”) as affected by variety and the processing method.

Variety	Processing method	Peak viscosity (RVU)	Trough (RVU)	Breakdown viscosity (RVU)	Final viscosity (RVU)	Setback viscosity (RVU)	Peak time (min)	Pasting temperature (°C)
UYF	Soak in water	82.69b	47.75b	34.94b	68.27b	20.52ab	6.97ab	72.65
	Parboil	115.90c	88.60c	27.29b	133.17c	44.56bc	8.70b	74.24
AOF	Soak in water	4.81a	4.33a	0.48a	6.23a	1.90a	4.30a	na
	Parboil	167.65d	119.90d	47.75bc	175.17d	55.27bc	5.45ab	50.69
NYF	Soak in water	161.50d	89.67c	71.21 cd	155.56 cd	68.60c	8.06b	77.91
	Parboil	231.67e	139.96d	92.25d	220.38e	80.13c	7.60ab	78.58
Effects								
Variety (V)		***	***	***	***	**	*	—
Processing method (P)		***	***	*	***	*	NS	—
V × P		***	**	*	**	NS	NS	—

Values followed by the same alphabet are not significantly different (*P* > 0.05). UYF, Ugandan Yellow-fleshed; AOF, American Orange-fleshed; NYF, Nigerian Yellow-fleshed; na (very low paste viscosities); NS, not significant.

^*^, ^*^^*^, ^*^^*^^*^Significant effects at*P* < 0.05, 0.01, 0.001 respectively.

### Correlations among properties of sweet potato flour (“elubo”)

The Hunter color parameters were more strongly correlated with protein, fat, fiber, and the carbohydrates which include starch and sugars with the correlations being negative forL*(Table6). The WAC, WS, and SWP also showed similar strong correlations although not with protein. The highest (*r* = 0.99) and significant (*P* < 0.01) correlations were observed between WS and sucrose or the total sugar.

**Table 6:** Correlations between chemical properties and functional properties of the traditional sweet potato flour (“elubo”).

Chemical property	L^*^	a^*^	*b*	WAC	WS	SWP
Moisture	−0.652*	0.196	0.092	0.361	−0.122	0.404
Protein	−0.906**	0.601*	0.597*	0.427	0.458	0.398
Fat	−0.932**	0.951**	0.908**	0.865**	0.801**	0.831**
Fiber	−0.676*	0.966**	0.953**	0.832**	0.933**	0.782**
Ash	−0.147	0.054	0.094	−0.283	0.101	−0.305
Carbohydrate	0.957**	−0.762**	−0.690*	−0.753**	−0.518	−0.746**
Starch	0.792**	−0.748**	−0.648*	−0.928**	−0.516	−0.932**
Amylose	0.226	−0.182	−0.037	−0.572	0.126	−0.640*
Fructose	−0.672*	0.792**	0.861**	0.349	0.874**	0.264
Glucose	−0.718**	0.771**	0.792**	0.712**	0.727**	0.677*
Sucrose	−0.578*	0.893**	0.948**	0.558	0.989**	0.476
Total sugar	−0.616*	0.906**	0.960**	0.578*	0.991**	0.498
pH	−0.203	0.326	0.253	0.335	0.217	0.322
TTA	−0.437	0.645*	0.758**	0.150	0.839**	0.055

WAC, water absorption capacity; WS, water solubility; SWP, swelling power; TTA, total titratable acidity;L^*^, whiteness;a^*^, redness;b^*^, yellowness.

* Correlation is significant at*P* < 0.05,

** Correlation is significant at*P* < 0.01.

All the pasting properties except peak time were significantly (*P* < 0.01) correlated (0.75 – 0.83) with moisture content of the sweet potato “elubo” (Table7). On the other hand, all the pasting properties showed negative correlations with the sugars. The most significant correlation was between total sugar and peak time (*r* = −0.805). TTA was also negatively correlated with the pasting properties and the correlation was significant (*P* < 0.05).

**Table 7:** Correlations between chemical properties and pasting properties of the traditional sweet potato flour (“elubo”).

Chemical property	Peak viscosity (RVU)	Trough (RVU)	Breakdown viscosity (RVU)	Final viscosity (RVU)	Setback viscosity (RVU)	Peak time (min)	Pasting temperature (°C)
Moisture	0.827**	0.752**	0.823**	0.793**	0.780**	0.071	0.827**
Protein	0.206	0.138	0.273	0.179	0.239	−0.418	0.206
Fat	0.048	0.130	−0.080	0.095	0.026	−0.610*	0.048
Fiber	−0.320	−0.183	−0.477	−0.257	−0.359	−0.724**	−0.320
Ash	0.037	0.009	0.077	0.038	0.074	−0.162	0.037
Carbohydrate	−0.391	−0.405	−0.316	−0.400	−0.349	0.427	−0.391
Starch	−0.351	−0.477	−0.115	−0.436	−0.321	0.263	−0.351
Amylose	−0.650*	−0.704*	−0.484	−0.637*	−0.446	−0.025	−0.650*
Fructose	−0.498	−0.513	−0.405	−0.501	−0.428	−0.743**	−0.498
Glucose	−0.198	−0.064	−0.372	−0.140	−0.236	−0.630*	−0.198
Sucrose	−0.616*	−0.527	−0.661*	−0.572	−0.588*	−0.797**	−0.616*
Total sugar	−0.590*	−0.501	−0.641*	−0.547	−0.566	−0.805**	−0.590*
pH	0.062	−0.002	0.153	0.015	0.031	−0.166	0.062
TTA	−0.738**	−0.737**	−0.637*	−0.731**	−0.642*	−0.712**	−0.738**

TTA, total titratable acidity.

* Correlation is significant at*P* < 0.05,

** Correlation is significant at*P* < 0.01.

The pasting properties were negatively correlated (*P* < 0.01) with WS than with other related functional properties (Table8).

**Table 8:** Correlations between functional and pasting properties of the traditional sweet potato flour (“elubo”).

Functional property	Peak viscosity (RVU)	Trough (RVU)	Breakdown viscosity (RVU)	Final viscosity (RVU)	Setback viscosity (RVU)	Peak time (min)
WAC	0.151	0.285	−0.070	0.218	0.080	−0.427
WS	−0.549	−0.462	−0.602*	−0.494	−0.498	−0.743**
SWP	0.234	0.372	−0.005	0.296	0.135	−0.388

WAC, water absorption capacity; WS, water solubility; SWP, swelling power.

* Correlation is significant at*P* < 0.05,

** Correlation is significant at*P* < 0.01.

### Consumer acceptability

**Figure 1:**
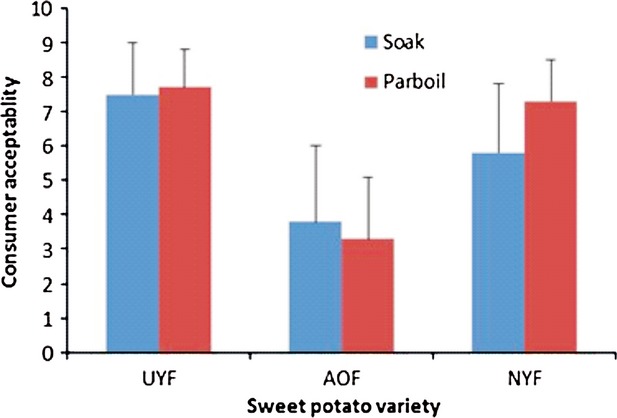
Consumer acceptability of sweet potato “amala” as influenced by variety and the processing method. UYF, Ugandan yellow-fleshed; AOF, American orange-fleshed; NYF, Nigerian yellow-fleshed.

Sensory scores for consumer acceptability of sweet potato “amala” are shown in Figure1. “Amala” from yellow-fleshed varieties were scored high (7.3–7.7) while those from the orange-fleshed variety were scored very low (3.3–3.8). Among the “amala” with high sensory scores, those prepared from sweet potato flour using the parboiling method were scored higher; Ugandan yellow-fleshed (7.7) and Nigerian yellow-fleshed (7.3). Acceptbility of “amala” was significantly correlated (*P* < 0.01) with ash,b* and WS (*r* ranged from −0.92 to −0.96), as well as with fiber, sugars, and peak time (*r* ranged from −0.83 to −0.91) (*P* < 0.05) of sweet potato “elubo”.

## Discussion

The moisture content of sweet potato “elubo” were higher than the range reported by Babajide et al. (2006) for traditional dry yam slices but lower than reported by Akissoe et al. (2001) for dry yam tubers and flour. Akissoe et al. (2001) had earlier reported that proximate composition (including moisture content) of dry yam tubers are similar to those of the flour except in crude fiber. The higher moisture content observed for sweet potato “elubo” from the Nigerian variety may be due to the thicker slices (50–60 mm) and sun-drying used to process this variety, as traditionally practiced. This is in line with the report of Babajide et al. (2006) that thicker slices (30–50 mm) and sun-dried yam chips had significantly higher moisture content than slices less than 30 mm and oven-dried. Falade and Solademi (2010) also reported that thinner slices of sweet potato showed lower moisture content and moisture ratio after drying. The authors attributed the lower moisture content of thinner slices to higher surface area relative to total area, exposed to heat during drying, as well as to lower diffusion pathway for vapor removal during drying. According to van Hal (2000), the moisture content of sweet potato flour is significant for storage, since water can accelerate chemical and microbial deterioration; the values reported for “elubo” in this study are within the range (10–13%) recommended for long shelf life (Christensen and Kaufmann 1973; Zhao and Jia 1985). The significant correlation between moisture content and the tristimulus colorL* was in accordance with the report of Shittu et al. (2007) for the cassava flour. According to the authors, the typical color charcteristic of flour is more conspicuous after reconstitution due to the higher moisture content. The correlation between moisture and pasting properties of sweet potato “elubo” suggests that the higher the moisture content, the higher the paste viscosities and pasting temperature.

The lower moisture values observed for the parboiling method for Ugandan yellow-fleshed variety may be due to varietal difference in properties of the starch granules; starch granules from different varieties of the same crop has been reported to behave differently. For example, Jangchud et al. (2003) reported that the starch granules of orange-fleshed sweet potato were smaller in size than those of the purple fleshed variety, and that this may have contributed to the differences observed in their paste viscosities.

The relatively high values of protein and fibers of “elubo” from orange-fleshed varieties were consistent with characteristics of flour from orange-fleshed sweet potato as reviewed by van Hal (2000). The lower fiber values of flour from the soaking method may be a confirmation that sweet potato is high in soluble fiber (WHO 1990; Kays and Kays 1997), which however may have leached out during soaking. This suggests that the soaking method may not be appropriate where the interest is to retainwater soluble components such as soluble fiber.

Amylose content of sweet potato flour was significantly affected by the variety and the processing method, although Akissoe et al. (2003) reported that only cultivar affected amylose component of starch in yam. Sucrose was the main sugar contributing to the total sugar in sweet potato “elubo”. Mestres et al. (2004) however reported that the main soluble sugar in yam “elubo” was glucose with minor amounts of fructose. The general sweetness of sweet potato over yam may be attributed to the fact that sucrose is sweeter than glucose (Gaman and Sherrington 1998). The lower values of sugar obtained for flour from the parboiling method is expected since solubility of sugars increase with increase in temperature, this may have been followed by leaching of sugars from the sweet potato slices into the parboiling water. The sweet potato flour had a lower pH and TTA value than that reported for yam flour (Kamenan et al. 1987; Akissoe et al. 2001). The significant negative correlation between TTA and pasting viscosities is a confirmation of the fact that the presence of acids affects gel strength as it reduces the paste viscosities in this study.

Sweet potato flour from the Ugandan yellow-fleshed roots which produced the most acceptable “amala” had a mean brown index of 1.84. Flour from the Nigerian yellow-fleshed variety produced by the parboiling method gave the next most acceptable “amala” and it had a brown index of 12.08. All the other flour types had a brown index of between 16.35 and 25.63. Mestres et al. (2004) reported a mean brown index of 19.8 for laboratory-prepared yam flour, while 21.0 was reported by Akissoe et al. (2001) for yam flour in Cotonou (Benin) markets. Although Mestres et al. (2004) also reported meana* andb* values of 1.7 and 10.1, respectively, the authors, however, reported that based on an earlier study, the brown index was the most representative color index because of the high significant correlation between the brown index of yam flour and that of the “amala”. Therefore, in line with these authors, although acceptable “amala” in this study were made from sweet potato flour with mean values ofa* (1.2) andb* (13.62), a brown index of between 1.84 and 12.08 may be suggested as appropriate for sweet potato flour for acceptable “amala”. Variety had a significant effect on the brown index but the processing method did not. This is in contrast with the report for yam (Akissoe et al. 2003). The significant difference in color parameters of the sweet potato flour must be due to differences in intrinsic properties of the varieties, such as the higher*β*-carotene content of the orange-fleshed variety.

The functional properties of flour are those that directly determine their end uses. It has been established that the composition and nature of macromolecules (proteins, fat, and carbohydrates) in food materials often affect their functionality (Prinyawiwatkul et al. 1997; Hung and Morita 2003). This was obvious in the several significant correlations between chemical components (particularly fat, fiber, starch, and sugars) in the sweet potato flour and the resulting WAC, WS, and SWP. The high significant correlations between fiber, sugars, TTA, and the WS reflect the highly soluble nature of these constituents thereby contributing positively to the WS of the flour. The higher the sucrose or total sugar as well as fiber, the higher the WS. Sweet potato is particularly rich in soluble fiber and sugars (Kays and Kays 1997). Prinyawiwatkul et al. (1997) reported that differences in SWP of starchy material can be attributed to starch content, presence of impurities, such as proteins and lipids, as well as pretreatment and processing history. The negative and highly significant correlations (*P* < 0.01) between pasting properties and sugars suggest the possibility of competition between starch and sugar for water, considering the fact that sugar content of sweet potato is relatively high. Nevertheless, this phenomenon has the advantage that only a small amount of flour will be required relative to the quantity of water to achieve significant swelling, this has implications for commercial purposes in terms of profit expectations. Parboiling increased the SWP of sweet potato flour. This is similar to the report of Jangchud et al. (2003) for blanching. Since parboiling is a process associated with increasing temperature compared to soaking in cold water, the trend for SWP in this study is also in agreement with Yadav et al. (2006). The marked variation in WAC, WS, and SWP of the sweet potato flour further establishes that intrinsic differences in biological properties as a result of varietal differences is a major factor in the utilization of sweet potato. The behavior of the orange-fleshed variety is particularly interesting in this regard. Furthermore, the observed wide variation in the functional properties of sweet potato flour in this study suggests the potential of finding diverse food utilization for sweet potato flour.

The temperature range over which gelatinization occurs is characteristic of the particular type of starch. The observed differences in paste viscosities among the sweet potato flour may be indicative of variation in the hydration and swelling capacities of their starches as reported by Henshaw et al. (1996) for cowpea flour from several varieties. However, in this study, there was no significant correlation between SWP and paste viscosities. The flour type from the orange-fleshed variety was particularly interesting when compared to the other two yellow-fleshed varieties in terms of the relatively high WAC, WS, and SWP. This may be an indication of the major influence of varietal differences. Also, the fact that flour from the orange-fleshed variety produced from soaking had low paste viscosities compared to the parboiling variety reflects the effect of the processing method.

## Conclusion

Yellow-fleshed sweet potato varieties were more appropriate for acceptable “amala”. Soaking and parboiling gave acceptable “amala”. This suggests that variety was a key factor affecting flour quality than the processing method in the production of sweet potato flour (“elubo”). Sweet potato flour with relatively lower values of fiber, ash,b*, sugars, and WS gave acceptable “amala”. Staple foods in Nigeria are characterized by a bland sour taste, it is, therefore, not surprising that the acceptable sweet potato “amala” was obtained from “elubo” with low sugars which was from the yellow-fleshed varieties used in this study.
